# Vesical Irritation in Women

**Published:** 1887-02

**Authors:** Virgil O. Hardon

**Affiliations:** Lecturer on Operative Gynæcology, Southern Medical College, Atlanta, Ga.


VOL. III. FEBRUARY, 1887. NO. 12.

### Original Communications.

### VESICAL IRRITATION IN WOMEN.*

BY VIRGIL O. HARDON, M. D.

LECTURER ON OPERATIVE GYNÆCOLOGY, SOUTHERN MEDICAL COLLEGE, ATLANTA, GA.

There is probably no class of diseases, outside of those which are from their nature necessarily incurable, which are ordinarily treated with so little satisfaction and success as the diseases of the female bladder and urethra. One reason for this may be found in the fact that the profession has heretofore had no means of making anything like a thorough physical exploration of these organs. As the treatment of uterine diseases was little more than mere guess-work and empiricism until a new impulse was given to it by Sims' invention of the speculum which bears his name, so until recently, for lack of the means of exploring by

* Read before the Atlanta Society of Medicine, November 16, 1886.

the eye and the touch the urinary tract of women, the treatment of diseases of that tract has been one of the *opprobria* of gynæcology. Another reason for failure in the treatment of derangements of the female bladder lies in a lack of appreciation of the causative effect of uterine disease in these affections. Many a woman has been faithfully and persistently treated for vesical irritation with frequent micturition by astringent injections into the bladder, when the whole cause of her trouble consisted of a displacement of the womb which produced a traction upon the vesical neck which could be remedied only by lifting the womb to its normal position in the pelvis. A very considerable proportion of all women who are the subjects of uterine derangement suffer more or less with bladder symptoms. This is not at all strange when we remember that the bladder derives its blood supply from the same arteries as the womb, that the venous connections of the two organs are most intimate, that its nerve fibres and those of the womb are inextricably interlaced, and that its connective tissue is continuous with that of the uterus. Between two organs so closely related in every way, it is evident that an intimate sympathy must exist and such is found to be the case in actual experience. Hence any treatment of irritation of the bladder which ignores existing uterine derangements will inevitably fail to accomplish a cure. At the same time, it is true that there are many cases in which irritation of the bladder has no dependence upon disease of the womb and the recognition of the presence or absence of such a dependence is the point upon which turns success or failure in the treatment.

Frequent micturition often exists in women when there is no other subjective symptom to indicate disease. These cases are sometimes seen in practice. But it is oftener the case that such patients mutely suffer for years, heroically bearing the pain and annoyance to which they are subjected with the patient endurance characteristic of their sex. Since there is no fixed law in regard to the normal frequency of micturition, the line which separates a healthy from a pathological condition is very indefinitely drawn and women often unconsciously overstep that line and may even pass far beyond it through lack of proper physiological teaching.

I have seen women who were compelled to urinate as often as every hour, yet who, in the absence of any other symptom, contented themselves with the idea that they had a "weak bladder," and instead of regarding it as a sign of disease, looked upon it as a constitutional peculiarity like a bad memory or a poor ear for music.

There is probably no symptom which, when existing to its fullest extent, is capable of producing more physical and mental anguish than the one under consideration. Pain, even though it be constant and severe, may be hidden by a calm exterior and a smiling face, so that none but the sufferer is aware of its existence. But the necessity of emptying the bladder at short intervals cannot be concealed or disregarded and debars the subject of it from the society of all except her most intimate friends of her own sex. Her finer sensibilities are shocked by the frequency with which her thoughts are compelled to revert to a function whose exercise, even under the most favorable circumstances, is but a disagreeable necessity. Continuous application to any pursuit, physical or mental, is rendered impossible by constant interruption. Change of scene, even for a short time, is prevented by the necessity of having ever at hand the conveniences for urination. The pleasures of society are forbidden, attendance upon religious service, that balm for many other afflictions, is equally denied, and nothing remains for the poor unfortunate but to become a prisoner in her room, oscillating between her lounge and her closet, and with a fate worse than that of Sisyphus, condemned to the one unending task of keeping her bladder empty. What wonder that under such circumstances life becomes an intolerable burden and death is looked forward to as a happy release. It was only a short time since that I was assured by a patient thus afflicted that nothing but a strong sense of duty restrained her from committing suicide.

The term frequent micturition is only a relative one, since within normal limits wide variation is possible. I have seen a woman who, being constantly employed in a public place during the day, emptied her bladder only at morning and night and yet suffered no inconvenience from the long retention of urine. In marked contrast with this case is one reported by Goodell of a lady who traveled a whole day in a stage-coach and from motives of delicacy failed to empty her bladder. When at her journey's end she could not pass her water and was obliged to call in a physician to draw it off. A chronic cystitis followed, which lasted for many years and which the author pronounces "the worst case he ever saw." The urine is normally discharged from the bladder five or six times during the twenty-four hours. But many women who are in perfect health, especially those who have borne children, urinate as often as every two hours during the day and are uncomfortable if compelled to retain the contents of the bladder for a longer time. On the other hand, many women whose occupation prevents so frequent micturition, such as teachers, saleswomen and the like, acquire the faculty of holding the urine for six or seven hours. I am convinced that habit has a strong influence in the matter and that a healthy bladder can be trained to submit to a much greater amount of habitual distention than is ordinarily required of it. The frequency of micturition is affected also by the total amount of urine excreted. This amount is on an average about thirty-five ounces in twenty-four hours, with variations ranging from twenty to forty-five ounces within the limits of health. In certain diseases the amount far exceeds this maximum and in such cases the frequency of micturition is of course correspondingly increased. Since no arbitrary line can be drawn which would in all cases separate the normal from the abnormal condition, frequent micturition can be defined only as micturition whose frequency causes inconvenience or annoyance and depends upon a morbid condition for its causation. Were it necessary to draw an arbitrary line, I would be inclined to select an interval of two hours as covering the majority of cases. But a more distinctive characteristic of abnormally frequent micturition is the necessity of habitually emptying the bladder at intervals during the night, a necessity which is never present when the organ is not subjected to morbid influences.

The sensation which leads to a desire for micturition originates in the circular muscular fibres within the bladder, especially the portion just posterior to the vesical termination of the urethra, sometimes spoken of as the vesical sphincter. As the bladder becomes distended by the accumulation of urine, such distention, when it has reached a certain point, is resisted by these circular muscular fibres. This muscular resistance gives rise to a desire to micturate by an impulse conveyed to the so-called "centre of micturition" in the lumbar portion of the spinal cord. Forcible mechanical expansion from any other cause will give rise to the same sensations and to spasmodic contractions of the bladder.

In addition to this mechanical mode of production of the desire for micturition, there is another set of influences, quite independent in their action, which produce the same result. This fact is well established both by physiological experiment and by pathological observation. Goltz has demonstrated that in dogs in whom the spinal cord has been divided in the dorsal region, normal and complete micturition may be excited by any slight stimulus, such as sponging the anus or pressing upon the abdominal walls. The same phenomenon is observed in man in cases of injury to the spinal cord and in children as the result of emotions or of irritation about the genitals. Fear has a potent influence in exciting the action of the "centre of micturition." Students at the commencement of an examination, and surgeons in the presence of a formidable operation, have often been seized with an irresistible desire to empty the bladder.

Hence, whenever a case of frequent micturition exists in a woman who presents none of the other symptoms of disease of the bladder, the explanation must be sought in some mechanical influence which produces traction upon the vesical neck or in some source of irritation in some other part of the body. In the former case it is not difficult to trace out the cause by proper and complete physical examination. But when the irritation is located in some more or less remote part and works through reflex action, it is by no means so easy to define it, and frequently it will be found very difficult or even impossible.

There are several varieties of small tumors which occur at and within the mouth of the urethra, which give trouble only by mechanical irritation. Most of these growths, if occurring elsewhere, would not be noticed. But in this situation they interfere with the free escape of urine and give rise to frequent desire for micturition through reflex irritation. If situated, as they frequently are, at or just within the meatus, they will scarcely escape detection. But if located within the urethra, the reflex symptoms may so completely mask the true condition that they will not be discovered. These growths may be pedunculated polypi, small neuromata or vascular growths. The last are the most common and are rarely found elsewhere than at the meatus. The neuromatous and pedunculated tumors may be found in any part of the canal and will often escape detection by ordinary methods of examination. No speculum has yet been invented by which can be gained a satisfactory view of the urethral canal for more than a very short distance beyond the meatus. For this reason the symptoms arising from the presence of such growths are often ascribed to other than their true causes. There exists, however, a means of diagnosis and treatment which has lately been introduced by Emmet, and which, although it has not yet been extensively adopted by the profession, has a great future. It fills a niche which has heretofore been empty and supplies a want which has been felt by every gynæcologist and which has been but very imperfectly filled by the devices of previous operators on the urethra. I refer to Emmet's "button-hole operation," which is fully described in the last edition of his Principles and Practice of Gynæcology. This operation consists in the establishment of an artificial urethro-vaginal fistula, through which the whole of the urethral canal can be thoroughly exposed and treated by topical applications when necessary.

When there is reason to suspect the existence of a small fibroid or neuromatous growth at a point within the urethra which is not accessible through the meatus, Emmet's button-hole operation should be performed for the detection and removal of such a tumor. When found, the growth should be snipped off with scissors and its base touched with acetic acid. The artificial fistula may then be immediately closed. If the tumor is located at or about the meatus, its removal in the same manner is a very simple affair. But care must be taken to remove as little as possible of the normal tissue, since cicatrization about the meatus is liable to lead to contraction and distortion of that orifice with disastrous results. When these growths are removed, they have very little tendency to return.

Prolapse of the urethral mucous membrane is sometimes met with as a cause of vesical irritation in women who have had frequent or long labors, from the pressing forward of the parts by the child's head. It is rarely, if ever, seen in nulliparous women. The mucous membrane projects from the meatus in a circular fold similar to the condition which exists in prolapse of the rectum. The prolapsed portion becomes irritated and excoriated and sometimes exceedingly painful. There is no difficulty in distinguishing this condition, as it is hardly possible to confound it with any other if an examination of the parts be made. The plan has been recommended of removing the prolapsed portion with knife or scissors. But I know of no procedure which should be more severely condemned. By such removal a permanent shortening of the urethra is produced, the neck of the bladder is drawn forward under the pubic bone and thus an incurable distortion of the parts is effected. Moreover, the cicatrization which follows the operation is liable to produce a contraction at the meatus which may lead to a chronic urethritis. The proper treatment consists in gently restoring the prolapsed mucous membrane to its normal position by means of a steel bougie by which it should be gradually pushed toward the bladder. This process may be aided by manipulation by a finger in the vagina. When the prolapsed canal has been wholly restored, the sound must not be drawn straight out for it would bring the mucous membrane with it. But by a rotatory, boring motion it may be removed without displacing the membrane. This procedure may require repetition many times, but in the end it will often effect a cure. When it fails, the most effectual treatment consists in restoring the mucous membrane as just described and, while the sound is *in situ*, performing the button-hole operation. The edges of the fistula should be brought together again with a portion of the urethral mucous membrane turned into the wound. In this way the membrane will be securely fastened in its normal position so that prolapse will be impossible.

In some cases a dilatation of the urethra occurs, forming a pouch which projects into the vagina and in which urine collects and is not discharged in urination. This constitutes a source of irritation which in time gives rise to vesical irritability and frequent micturition. It is usually caused in the same way as prolapse of the urethra, that is, by the forward pressure of the fœtal head during labor. A pouch is formed capable of holding from one to two drachms which may be readily detected by digital or ocular examination through the vagina. A sound passed into the urethra while a finger is in the vagina will make the condition more obvious and will facilitate its diagnosis from a tumor of the urethro-vaginal septum. For this condition there is only one method of treatment which is attended by satisfactory results. A portion of the septum must be removed of such size as to restore the canal to its normal calibre. This may be done by passing a large-sized sound into the urethra and then taking up with forceps a fold of the septum of sufficient extent to draw the tissues closely about the sound. This fold will constitute the portion to be removed and such removal should be done with curved scissors. The edges of the wound should then be united with sutures and primary union will readily occur.

Stricture of the urethra may occur in the female as in the male. It is not as common, however, does not entail as serious consequences and is more readily cured. It consists of a cicatricial thickening of the mucous membrane by which the calibre of the canal is diminished. It may be caused by gonorrhœal inflammation, by mechanical injury, as in labor, for instance, or by improper use of caustic applications. It may occur at any point in the canal, but is more frequent at or near the meatus, since that is the portion of the canal which is most exposed to the exciting causes. Vesical irritation and abnormal frequency of micturition constitute the most prominent symptoms. The finger in the vagina may feel the hard, gristly cicatrix at the meatus or through the urethro-vaginal septum, while a sound passed into the urethra will detect the stricture by the difficulty experienced in passing it.

These strictures are usually susceptible of ready cure by gradual dilatation by bougies as in the male. Forcible divulsion and division with the knife are not to be recommended, since the wounds thus produced only increase the amount of cicatricial tissue, and hence the liability to subsequent contraction. If the stricture be at the meatus and the orifice be very much contracted and surrounded by cicatrices, it is sometimes preferable to perform the button-hole operation and leave the fistula permanently open.

At this point permit me to say a word about forcible dilatation of the urethra as a means of diagnosis and of treatment. This procedure has been advocated by many eminent authorities who report good results from it. There is no reason to doubt that their results were as good as they have represented. But my experience and observation lead me to believe that in the hands of the average practitioner the operation is not one to be recommended on account of the liability to the production of permanent incontinence of urine. I have seen such incontinence occur repeatedly in the practice of men who were neither careless nor unskillful. As long as this constituted the only method of obtaining access to the urethral canal, it became necessary to resort to it at times when digital exploration was imperative. But since Emmet has shown a way which is simple, easy and free from all danger of unpleasant after-effects, forcible dilatation should be consigned to the limbo of forgotten things. The physician should never be tempted to thrust his finger through a urethra into a bladder under the impression that it is simpler or safer than the surgical procedure which I have mentioned, for if he does, he may regret it when it will be too late for regret to avail him anything and a woman doomed to a lifetime of urinary incontinence may blast his reputation and destroy his peace of mind.

But the most common mechanical cause of vesical irritation consists of a downward displacement of the womb. In the textbooks it is stated that the pressure of a dislocated womb upon the bladder produces irritation of that viscus. But a moment's reflection will serve to show the incorrectness of this statement. The normal position of the womb is that of anteflexion so that the fundus rests upon the bladder. In addition to this, the bladder is always subjected to more or less pressure from the weight of the super-imposed viscera which rest upon it and follow it down as it contracts during micturition. Hence the bladder is always subjected to a greater or less amount of pressure, and if pressure could produce vesical irritation, no woman would ever be free from this symptom. In any event, the additional weight of a displaced uterus, which normally weighs only one and a half ounces, could not produce any appreciable effect. It is not the pressure of the womb, but the traction of that organ upon the neck of the bladder that produces the frequent desire for micturition in such cases. The anatomical relation of the parts shows this. It is also shown by the results of treatment. In the majority of cases of frequent micturition in women in whom no organic disease of the bladder or urethra exists, the symptoms are relieved by raising the womb in the pelvis and keeping it raised by appropriate mechanical means.

Sometimes the cervix uteri will be found lying far back in the pelvis, within the hollow of the sacrum and pressing upon the rectum. This may be due to a rotation of the whole uterus upon its transverse axis, to a shortening o fthe utero-sacral ligaments, or more rarely to a retroflexion of the cervix. Whatever its causation, the effect is to produce traction upon the bladder through the so-called utero-vesical ligament. In such cases a finger in the vagina will feel a tense band of tissue extending from the cervix forward to the bladder. With this condition, vesical irritation and frequent micturition are the rule, their absence the exception. There is usually a feeling of constant desire for micturition even when the bladder is empty. If the cervix be brought forward into its normal axis in the vagina this sensation immediately disappears as if by magic. It disappears because the traction upon the bladder ceases.

Hence there are usually two indications to be met in cases where vesical irritation is produced by traction of the womb upon the bladder, viz., to keep the womb at its normal level in the pelvis and to keep the cervix in its normal axis in the vagina. Any plan of treatment which meets these two indications will produce satisfactory results. I know of no device which accomplishes the purpose as completely as does the supporting tampon. The pledgets of cotton surrounding the cervix may be so graduated as to restore it to its normal axis, while the larger underlying pad raises the womb as a whole to its normal position in the pelvis.

But while the supporting tampon gives immediate and complete relief in such cases, it must not be regarded as a finality. The physician who stops at this point will have failed to cure his patient. Behind the displacement of the womb is some pathological change which has added to the weight of the organ either by an increase of the amount of blood contained in its tissues, or by an increase of its solid constituents, or by both combined. The condition which sets in motion the train of causes which eventuates in vesical irritation may be a stenosis of the cervical canal, a rupture of the perineum, a laceration of the cervix or any other factor capable of producing congestion, causing hypertrophy or arresting involution. A radical and permanent cure involves, therefore, the removal of the prime cause, whatever it may be. In other words, vesical irritation may be relieved in this class of cases by the supporting tampon, but it can be permanently cured only by the cure of the co-existing uterine disease.

When no organic disease of the pelvic organs exists and there is no displacement of the womb, the case may be regarded as one of purely reflex irritation and its source must be sought out if possible. It may be in the kidneys, the ureters, the external organs of generation, the anus, the rectum, the spinal cord or any of the nerve centres throughout the body. Hysteria, that mysterious affection of protean shape, may give rise to frequent micturition. It is necessary to seek out the abnormal condition and remedy it by the proper therapeutic or operative means. It would be outside the scope of this paper to consider the treatment of these various affections. The point which I would especially insist upon is, that in a case of irritable bladder, manifested by frequent micturition, it must not be taken for granted that the seat of disease is in the bladder. Many a patient has been dosed with buchu, cubebs, nitre, citrate of potash and the like for a supposed cystitis, when a simple examination would have revealed a fissure of the anus, a stricture of the urethra or a displacement of the womb as the cause of all the trouble. If the possibility of such an origin of frequent micturition be borne in mind, one will not be apt to be led into error, for the differential diagnosis will be easy. It is only by ignorance or forgetfulness of such a possibility that the physician is liable to be led astray.

I have selected a few typical cases from a larger number which have come under my observation, to illustrate the views contained in this paper. In these cases the relation of cause and effect is unmistakable. Often it is not so apparent and can be discovered only by careful and thorough investigation.

CASE I.—A. S., aged 22, married three years, sterile. Dysmenorrhœa since puberty, aggravated by marriage. About a week before each period begins to suffer from frequent desire for micturition which increases until, at the appearance of the flow, she is obliged to urinate about every half hour, day and night. With the cessation of the menstrual flow, the vesical irritation ceases. Bladder and urethra healthy. Womb in normal position. Cervix one and three-quarter inches long, extremely conoidal. One-half an inch of the cervix was amputated and the cervical canal dilated to the extent of one inch. Complete and permanent relief of dysmenorrhœa and frequent micturition.

CASE II.—S. B., aged 37, married, three children and one miscarriage. Dragging sensation in the loins, pain in back and pelvis extending down the thighs. Defecation very painful. Urinates hourly during the day and two or three times during the night. No disease of bladder or urethra. Slight unilateral laceration of cervix. Womb congested and low in the pelvis. Fissure of the anus extending upward from the cutaneous border for a third of an inch. The anus was thoroughly stretched. The fissure healed and the frequency of micturition entirely ceased.

CASE III.—F. T., married, aged 44, stupid and ignorant negress. Frequent micturition of ten days standing with occasional incontinence. Bladder, urethra and uterus healthy. Rectum filled with hard fæces. Had had no operation for thirteen days. Cathartics were used without effect. The rectum was therefore emptied by means of the fingers, a spatula and injections of warm water. As soon as the rectum was unloaded the bladder trouble ceased.

CASE IV.—(Reported in THE JOURNAL, February, 1886.) E. G., colored ,single aged about 30. Fell upon the buttocks three years ago. Pain in back and loins, difficulty of locomotion, dysmenorrhœa, painful defecation, incontinence of urine. Bladder and urethra healthy. Marked retroflexion. The womb was restored daily and supported by a tampon. At the end of a month the incontinence had entirely ceased and patient was well.

CASE V.—A. D., aged 28, single. Frequent micturition and occasional incontinence for four months. Urine normal, bladder healthy. Three-quarters of an inch above the meatus a tumor as large as a pigeon's egg in the urethro-vaginal septum. This was excised and found to be a sac communicating with the urethra by a minute opening. The relief of the bladder symptoms was permanent and complete.

